# Association of Dietary Micronutrient Intake with Pulmonary Tuberculosis Treatment Failure Rate: ACohort Study

**DOI:** 10.3390/nu12092491

**Published:** 2020-08-19

**Authors:** Ke Xiong, Jinyu Wang, Jianwen Zhang, Haibo Hao, Qiuzhen Wang, Jing Cai, Aiguo Ma

**Affiliations:** Institute of Nutrition and Health, School of Public Health, Qingdao University, Qingdao 266071, China; kexiong@qdu.edu.cn (K.X.); wangjinyu@qdu.edu.cn (J.W.); zhangjianwen_food@126.com (J.Z.); Haibofood@163.com (H.H.); kevin_1971@126.com (Q.W.); jingfox@163.com (J.C.)

**Keywords:** micronutrient, tuberculosis, diet, vitamin C, zinc

## Abstract

Malnutrition is associated with an increased risk of pulmonary tuberculosis (PTB) treatment failure. Currently, there is no effective adjunctive nutritional therapy. The current objective is to investigate the association of dietary micronutrient intake with PTB treatment outcome.A cohort study including 1834 PTB patients was conducted in Linyi, China. The dietary micronutrient intake was assessed through a three-day 24 h dietary recall questionnaire. The treatment outcome was assessed by combinations of sputum smear and computerized tomography results. A multivariate binary regression model was used to assess the associations. The final model was adjusted for potential confounding factors. A low intake of vitamin C (adjusted OR (95% CI): 1.80 (1.07, 3.04), P_trend_ = 0.02) and Zn (adjusted OR (95% CI): 2.52 (1.25, 5.08), P_trend_ = 0.02) was associated with a high treatment failure rate. In addition, a low intake of vitamin C and Mn was associated with a severe tuberculosis symptom, as indicated by a high TB score. A supplementation of vitamin C and Zn may be beneficial in PTB treatment. Previous meta-analysis of randomized controlled trials (RCTs) reported a null effect of Zn supplementation on PTB treatment. The effect of vitamin C supplementation should be investigated by RCTs.

## 1. Introduction

Tuberculosis is an infectious disease caused by *Mycobacterium tuberculosis*. The World Health Organization estimated 10.00 million tuberculosis incidences, and 1.49 million tuberculosis-related deaths worldwide in 2018 [1]. China has a high tuberculosis burden, which had0.87 million tuberculosis incidences and 39.40 thousand tuberculosis-related deaths in 2018 [1]. Tuberculosis remains the deadliest infectious disease worldwide [1]. Pulmonary tuberculosis is the major type of tuberculosis, which accounts for 85% of the total cases [2].

Malnutrition plays an important role in the treatment failure of pulmonary tuberculosis. Using body-mass index (BMI) as an indicator of nutritional status, Hoyt et al. showed that a BMI < 16 kg/m^2^ was associated with an increase in the number of cavity and affected lung zones in pulmonary tuberculosis patients [3]. Malnutrition is closely correlated with an impaired immune system, which weakens the patient’s ability to defend against mycobacteria [4]. Particularly, deficiency in micronutrients contributes to the impairment of an immune system through several mechanisms. For example, vitamin D deficiency could reduce the phagocytosis of macrophages [5], as well as the production of antibacterial peptides like cathelicidin [6], which are important for the intracellular killing of mycobacteria. On the other hand, Zn and Cu deficiency limited the killing of mycobacteria through the mechanism of metal poisoning in phagosomes [4].

Dietary intake is an important factor in modulating the nutritional status of pulmonary tuberculosis patients, and may ultimately affect the outcome of pulmonary tuberculosis treatment. Previous studies reported the association between dietary intake and the infection risk of pulmonary tuberculosis [7,8,9]. However, no effective adjunctive nutritional therapy is available for the treatment of pulmonary tuberculosis treatment. Dietary micronutrients intake was shown to be important for human health [10,11]. Particularly, vitamin C has pleiotropic effects on human health, possibly due to its antioxidant properties and cofactor functions for numerous biosynthetic enzymes [12]. In addition, vitamin C is widely considered to be important for optimal immunity [13]. On the other hand, Zn is involved in many biological functions, including immune responses, oxidative stress responses, DNA replication and damage repair, apoptosis etc., [14]. However, the precise role of many micronutrients like vitamin C on tuberculosis treatment is undetermined.

In this work, we conducted a prospective cohort study to investigate the associations between the intake of micronutrients from patients’ normal diet and the outcome of pulmonary tuberculosis treatment. We used a multivariate logistic regression model to adjust for other important factors for pulmonary tuberculosis treatment, including age, BMI, gender, smoking, drinking, occupation, newly or relapsed tuberculosis status, presence of lung cavity, diabetes and exposure to sunshine, as well as intake of vitamin A, vitamin C, vitamin E, Zn, Mg and Cu.

## 2. Materials and Methods

### 2.1. Ethics

The study was conducted in accordance with the Declaration of Helsinki. The study was registered on the Chinese Clinical Trial Registry (registration number ChiCTR-OCC-10000994), and approved by the Medical Ethics Committee of Qingdao Disease Prevention and Control Center (2009-4). All participants provided informed consent.

### 2.2. Study Design and Population

A cohort study was conducted between 2009 and 2013. Participants were recruited from the tuberculosis clinics, which were located in Linyi City, Shandong Province, China. The eligibility criteria include: (1) diagnosed as having pulmonary tuberculosis based on combinations of sputum smear, computed tomography (CT) results and clinical symptoms, referring to the Chinese national tuberculosis prevention and control guideline (2008) (abbreviated as “Chinese National Guideline” for the following text) [15]; (2) more than 18 years old; (3) providing informed consent. Exclusion criteria include: (1) having diseases like gastritis, which affect eating; (2) having serious complications, such as cardiovascular and lung complication, cancer or HIV; (3) having mental illness or impaired cognitive function; (4) having micronutrient supplementation in the past two months; (5) multi-drug-resistant tuberculosis; (6) pregnant or lactating women.

### 2.3. Procedure

At entry to the hospital, the participants received a standard questionnaire about their demographic characteristics, including age, gender, education, habit (including smoking and drinking etc.) etc. Two weeks after starting the antituberculosis treatment, the participants were surveyed by a standard 24 h dietary recall questionnaire by our project members for three days, including two weekdays and one weekend. The methodology refers to the Chinese Health Industry Standard WS/T 426.1-2013, and was validated before [16]. Briefly, the respondents were asked to recall their food intake, including portion size, in the past consecutive three days. The investigators were trained to be familiar with the commonly consumed food and its portion size in Linyi, and use a standard questionnaire to record the results. The dietary micronutrient intake was then calculated by referring to the standard China Food Composition Tables [17]. A standard questionnaire was used to assess the clinical characteristic of the tuberculosis patients, including cough, sputum production, hemoptysis, chest pain, fatigue, night sweating, fever and loss of appetite.

TB score was used as a comprehensive index to assess the clinical symptoms of the tuberculosis patients, as reported previously [18]. Having any of the eight mentioned symptoms above would give one point; having a BMI equal to or less than 16 kg/m^2^ would give two points, BMI between 16 kg/m^2^ and 18 kg/m^2^ would give one point, and BMI more than 18 kg/m^2^ would give zero points. Therefore TB score would range from 0 to 10. A higher TB score indicates a more severe clinical symptom. The height and weight of the participants were measured by our project members. And BMI was calculated by the following formula: BMI = weight [kg]/(height [m])^2^.

We worked with the staff from the tuberculosis clinics to follow up on the tuberculosis treatment of the participants. The follow-up periods ended at the end of the treatment. The participants received standard tuberculosis treatment according to the Chinese National Guideline [15]. Briefly, the patients received isoniazid, rifampicin, pyrazinamide and ethambutol for the first two months, and isoniazid and rifampicin for the following four months. The cure criteria were: the patients received all treatments and had two consecutive negative sputum smear results, one of which was obtained at the end of the treatment; CT results showed no active lesions.

### 2.4. Variables

The primary outcome was the failure rate of pulmonary tuberculosis treatment among the participants. The secondary outcome was the TB scores at the end of Week 2 antituberculosis treatment. The primary outcome was prespecified. The predictors were the micronutrient intakes of the participants according to their 24 h dietary recall survey. Potential confounding factors were: age, gender, BMI, smoking, drinking, occupation, newly or relapsed tuberculosis, presence of lung cavity, diabetes and exposure to sunshine. The TB score results were divided into two groups with a close number of participants (TB score ≤ 3 or TB score > 3 group). The micronutrient intakes were divided into three categories with a close number of participants. The highest tertile of micronutrient intake was used as the reference category.

### 2.5. Data Quality Control

The participating staff from the tuberculosis clinics all attended trainings by the same project team members about study objective, method and matters to consider. The filled survey was checked by at least one other project member. The data recording was performed by two project members, and they weredouble checked against each other.

### 2.6. Statistical Analysis

The statistical difference for the characteristics and micronutrient intakes between the treatment success and the treatment failure group was tested by a Mann–Whitney U test for non-normal data, a t-test for normal data, and a χ^2^ test for categorical data. The association between the dietary micronutrient intake and the failure rate of pulmonary tuberculosis treatment (or TB score) was assessed by a binary logistic regression model, with both univariate and multivariate analysis. In the multivariate analysis, the model was adjusted for potential confounding factors, including age, gender, BMI, smoking, drinking, occupation, newly or relapsed tuberculosis status, presence of lung cavity, diabetes and exposure to sunshine. The multivariate regression model was also adjusted for a few micronutrient intakes, which were claimed relevant by previous literature (including vitamin A, vitamin C, vitamin E and Zn) [7,19,20], or significantly different between the treatment success and the failure group (Mg and Cu, as shown in Table 1).

Linearity between the continuous independent variables and the dependent variables was tested by the Box–Tidewell method [21]. Briefly, the interactions of the continuous independent variables and their natural logarithm conversion values were included in the binary regression model to test significance. Insignificance means linearity. Collinearity among the independent variables was tested by the collinearity statistics in the SPSS software, including tolerance and variance expansion factor (VIF). Tolerance < 0.1 or VIF > 10 means that collinearity exists. The linear trend for the micronutrient variables was tested by using the median values of each category, and continuous variableswere included in the logistic regression model. P < 0.05 was considered statistically significant.

## 3. Results

**Figure 1:**
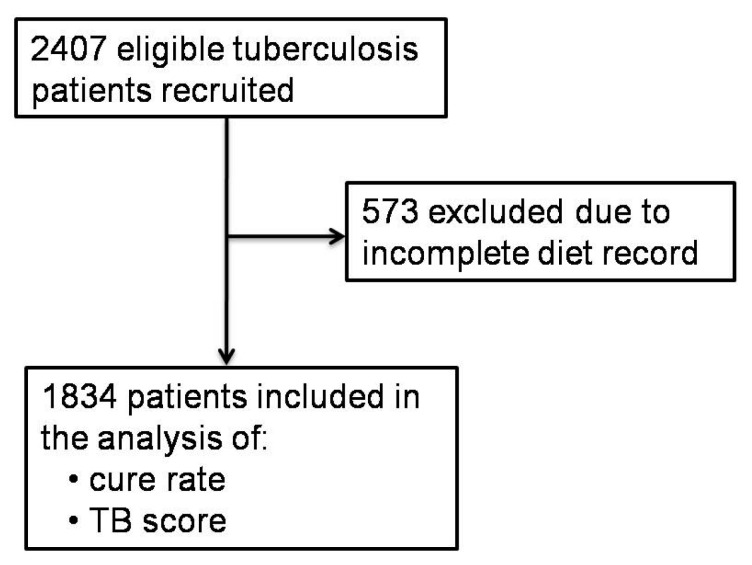
Trial flow.

As shown in Figure 1, 2407 eligible pulmonary tuberculosis patients were recruited between 2009 and 2013, from Linyi City, Shandong Province, China. A total of 573 patients were excluded from the study, due to an incomplete record for the three-day 24-h dietary recall survey. The remaining 1834 patients were included in the analysis. The included patients were followed up for an average of 6 months.

Table 1 displays the basic demographic characteristics at study entry and dietary micronutrient intake of the included 1834 pulmonary tuberculosis patients, after two weeks’ antituberculosis treatment. The age, occupation, newly or relapsed disease status and exposure to sunshine were significantly different between the treatment success and the treatment failure group. The treatment failure group had a higher median age, more farmers, more patients with relapsed tuberculosis and more patients with <40 min per day exposure to sunshine. In addition, the treatment failure group had significantly more intakes of dietary Mg and Cu. The rest of the independent variables were similar between the treatment success and the treatment failure group. The patients had a widely inadequate intake of dietary micronutrient intake, according to the Chinese Dietary Reference Intakes (Table 1) [22].

Table 2 displays the association between the dietary micronutrient intake and the failure rate of the pulmonary tuberculosis treatment by a binary logistic regression model. In the univariate analysis, no dietary micronutrient intake showed a significantly increased risk for the failure of pulmonary tuberculosis treatment. In the multivariate analysis, compared with the corresponding higher percentiles, the lowest percentiles of vitamin C (adjusted OR (95% CI): 1.80 (1.07, 3.04), P_trend_ = 0.02) and Zn (adjusted OR (95% CI): 2.52 (1.25, 5.08), P_trend_ = 0.02) intake were associated with an increased failure rate of pulmonary tuberculosis treatment, while both the lowest (adjusted OR (95% CI): 0.33 (0.14, 0.79), P_trend_ = 0.01) and middle percentiles (adjusted OR (95% CI): 0.50 (0.28, 0.90), P_trend_ = 0.01) of Mg intake showed a decreased risk for the treatment failure. In addition, the higher age, relapsed tuberculosis and <40 min/d exposure to sunshine showed a significantly increased risk for the failure of pulmonary tuberculosis treatment.

The association between the dietary micronutrient intake and the severity of tuberculosis symptoms (TB score) was shown in Table 3. In the univariate analysis, compared with the highest percentile intake, the lowest percentile of vitamin C (OR (95% CI): 1.90 (1.52, 2.39), P_trend_ < 0.001), Ca (OR (95% CI): 1.48 (1.18, 1.86), P_trend_ = 0.001), Mg (OR (95% CI): 1.41 (1.13, 1.77), P_trend_ = 0.004) and Mn (OR (95% CI): 1.50 (1.20, 1.88), P_trend_ < 0.001) intake was associated with a significantly increased TB score. The middle percentile of vitamin C and Mn intake also showed a significantly increased TB score.

In the multivariate analysis, the lowest and middle percentile of vitamin C, Mg and Mn intake showed a significantly increased TB score. In addition, a higher age, excessive drinking, relapsed tuberculosis and <40 min/d exposure to sunshine were also associated with a significantly higher TB score; while a higher BMI was associated with a significantly lower TB score.

## 4. Discussion

Malnutrition is associated with the failure of pulmonary tuberculosis treatment. However, no effective adjunctive nutritional therapy exists for pulmonary tuberculosis treatment. We investigated the association of dietary micronutrient intake with the failure rate of pulmonary tuberculosis treatment, and found that the low intake of vitamin C and Zn could contribute to the increased failure rate. We also found that the low intake of vitamin C and Mn may increase the severity of tuberculosis symptoms.

Vitamin C was shown to induce reactive hydroxyl radicals in vitro, through the Fenton reaction in a ferrous-ion-dependent manner. The induced hydroxyl radical can sterilize drug-susceptible and drug-resistant *Mycobacterium tuberculosis* [23]. Subsequent murine experiment showed that vitamin C can improve the killing of *Mycobacterium tuberculosis* by the first-line tuberculosis drugs, including isoniazid and rifampin [24]. In addition, two human studies indicated that dietary vitamin C intake was associated with a reduced infection risk of pulmonary tuberculosis [7,9]. Our results further support these by showing that the lowest tertile of dietary vitamin C intake can significantly increase the failure rate of pulmonary tuberculosis treatment, versus the highest tertile(adjusted OR (95% CI): 1.80 (1.07, 3.04), P_trend_ = 0.02). In addition, the lowest percentile (OR (95% CI): 1.49 (1.14, 1.96), P_trend_ = 0.002) and the middle percentile (OR (95% CI): 1.32 (1.02, 1.71), P_trend_ = 0.002) of vitamin C were associated with a more severe tuberculosis symptom, as indicated by a significantly increased TB score. The effects of vitamin C on pulmonary tuberculosis treatment should be further evaluated by a randomized controlled trial.

No association was observed between the outcome of pulmonary tuberculosis treatment and other vitamins, including vitamin A, thiamin, nicotinic acid, riboflavin and vitamin E. Consistently, previous randomized controlled trials showed null effects of both vitamin A [25,26] and vitamin E on pulmonary tuberculosis treatment [27].

Elevated Zn level in phagosomes are expected to kill mycobacteria via metal poisoning [4,28]. In addition, Zn is vital in thymus function, which modulates the maturation of T lymphocyte [20]. Our results indicate an inverse association of dietary Zn intake with the treatment failure rate of pulmonary tuberculosis (adjusted OR (95% CI): 2.52 (1.25, 5.08), P_trend_ = 0.02). However, a recent meta-analysis of previous randomized controlled trials indicated a negative effect of Zn supplementation on pulmonary tuberculosis treatment [29]. The association between the low intake of micronutrients and the high failure rate of pulmonary tuberculosis treatment does not prejudge a positive effect of micronutrient supplementation [30]. A randomized controlled trial should be used to confirm the effect.

Our results also show that an increased age, relapse and a low exposure to sunshine were the risk factors for the treatment failure of pulmonary tuberculosis. Relapsed patients are susceptible to multi-drug-resistant tuberculosis, which significantly increase the challenge for treatment. On the other hand, the exposure to sunshine is closely correlated with the synthesis of vitamin D in the human body [31]. Vitamin D was shown to promote the immune system for defending mycobacteria [5,6]. Consistently, the treatment failure rate of pulmonary tuberculosis was increased by a low exposure to sunshine (adjusted OR (95% CI): 1.88 (1.17, 3.02), P_trend_ = 0.009).The dietary intake of vitamin D is minimal in Linyi City. Fish and fish products are not regularly consumed by the residents, because Linyi is an inland city.

The strengths of the study are as below. First, the 24 h dietary recall survey was conducted for three days, including two weekdays and one weekend, and the average was used to calculate the dietary nutrient intake. This reduced the risk of selection bias with regard to the day-to-day and the weekday, versus the weekend variation for the diet selection of the participants. Second, a comprehensive index, TB score, was used to indicate the severity of the tuberculosis symptoms of each participant, which should be complementary to the results of treatment outcome. Third, we tried to collect data on all potential confounding factors for pulmonary tuberculosis treatment, and adjusted for the factors, with significant differences between the successful and the failed treatment groups in the multivariate regression model.

The limitations of the study should be acknowledged. First, the cohort study was conducted in Linyi City, which is a less developed inland city in Shandong Province, China. The surveyed dietary micronutrient intake may not be representative for other more developed cities or coastal cities. Second, the serum micronutrient status of the participants was not measured due to a limited budget. The absorption of micronutrients may be affected by factors such as genotypes, the intake of other food components, disease status etc. [32] Future work should focus on determining the serum micronutrient status of the tuberculosis patients and its association with the cure rate.

## 5. Conclusions

In conclusion, adequate vitamin C and Zn may be beneficial for increasing the cure rate of pulmonary tuberculosis patients. Previous meta-analysis of randomized controlled trials showed a null effect of Zn supplementation on pulmonary tuberculosis treatment. Future research may evaluate the effects of vitamin C on the treatment of pulmonary tuberculosis by a randomized controlled trial design.

## Figures and Tables

**Table 1:** Basic characteristics at study entry and dietary micronutrient intake after two weeks’ antituberculosis treatment for the included 1834 tuberculosis patients.

	Successful (*n* = 1710) ^1^	Failed (*n* = 124) ^1^	P ^2^	Number of Patients with Insufficient Micronutrient Intake (Percentage) ^3^
Age	55.0 (28.0)	59.5 (29.0)	0.002	-
Gender (male)	1357 (79.4%)	91 (73.4%)	0.12	-
BMI (kg/m^2^) ^4^	20.7 (2.7)	21.1 (2.7)	0.13	-
Smoking	467 (27.3%)	34 (27.4%)	0.98	-
Excessive drinking	601 (35.1%)	42 (33.9%)	0.77	-
Education			0.1	-
Primary school and below	971 (56.8%)	81 (65.3%)		
Middle and high school	701 (41.0%)	39 (31.5%)		
High education	38 (2.2%)	4 (3.2%)		
Occupation (farmers)	1602 (93.7%)	122 (98.4%)	0.03	-
Presence of lung cavity	230 (13.5%)	16 (12.9%)	0.86	
Newly diagnosed (newly)	1571 (92.4%)	100 (82.6%)	<0.001	-
Anemia	13 (0.8%)	2 (1.6%)	0.58	-
Hypertension	54 (3.2%)	5 (4.1%)	0.59	-
Diabetes	37 (2.2%)	5 (4.0%)	0.18	-
Exercise (adequate)	970 (56.8%)	64 (51.6%)	0.26	-
Exposure to sunshine (>40 min)	1463 (85.6%)	96 (77.4%)	0.01	-
Total energy intake (kcal/d)	1572.1 (766.4)	1615.4 (775.3)	0.51	-
Vitamin A (ug RAE/d) ^5^	229.5 (317.0)	245.7 (352.6)	0.57	1690 (92.1%)
Thiamine (mg/d)	1.0 (0.7)	1.1 (0.7)	0.3	1276 (69.6%)
Nicotinic acid(mg NE/d) ^5^	11.4 (6.7)	11.2 (6.5)	0.47	1278 (69.7%)
Riboflavin (mg/d)	0.6 (0.4)	0.6 (0.3)	0.15	1775 (96.8)
Vitamin C (mg/d)	50.0 (60.5)	40.0 (63.0)	0.15	1492 (81.4%)
Vitamin E (mg α-TE/d) ^5^	11.2 (10.4)	13.7 (13.3)	0.07	1147 (62.5%)
K (mg/d)	1464.5 (789.6)	1539.4 (814.6)	0.33	1471 (80.2%)
Na (mg/d) ^6^	422.8 (866.1)	407.6 (812.5)	0.26	1517 (82.7%)
Ca (mg/d)	269.7 (199.5)	303.5 (223.9)	0.09	1824 (99.5%)
Mg (mg/d)	290.6 (169.4)	315.5 (194.0)	0.05	1112 (60.6%)
P (mg/d)	951.3 (481.0)	978.0 (580.3)	0.15	431 (23.5%)
Fe (mg/d)	17.5 (9.4)	18.6 (9.7)	0.12	434 (23.7%)
Zn (mg/d)	8.6 (4.6)	9.2 (5.0)	0.14	1369 (74.6%)
Cu (mg/d)	1.7 (1.0)	1.9 (1.1)	0.02	155 (8.5%)
Se (μg/d)	37.4 (22.3)	39.6 (22.9)	0.88	1631 (88.9%)
Mn (mg/d)	5.7 (3.6)	6.3 (3.8)	0.06	-

^1^ Numerical variables are presented as median (IQR) unless noted otherwise; categorical variables are presented as number of patients in a specific category (percentage). ^2^ The statistical difference for the non-normal data was tested by a Mann–Whitney U test; the difference for the normal data was tested by a t-test; the difference for the categorical data was tested by a χ^2^ test. ^3^ The sufficiency of the micronutrient intake was checked against the Chinese Dietary Reference Intakes (reference 22). ^4^ BMI values are presented as mean (SD). Furthermore, there was n = 1708 for the successful group, and n = 123 for the failed group. ^5^ 1 ug RAE = 1 ug all-trans-retinol + (1/12) ug all-trans-β-carotene + (1/24) ug provitamin A carotenoid; 1 mg NE = 1 mg niacin + (1/60) mg tryptophan; 1 mg α-TE = 1 mg α-tocopherol + 0.5 mg β-tocopherol + 0.1 mg γ-tocopherol + 0.02 mg δ-tocopherol + 0.3 mg α-tocotrienol. ^6^ The intake of Na as salt was not counted.

**Table 2:** Association of the dietary micronutrient intake with the failure rate of tuberculosis treatment.

	Group	Univariate			Multivariate ^2^		
		OR (95% CI)	P	P_trend_	OR (95% CI)	P	P_trend_
Age	-	1.02 (1.01, 1.03)	0.003	-	1.01 (1.00, 1.03)	0.04	-
Gender ^1^	-	0.72 (0.47, 1.09)	0.12	-	0.67 (0.42, 1.07)	0.09	-
BMI	-	1.05 (0.99, 1.12)	0.13	-	1.06 (0.99, 1.13)	0.10	-
Smoking ^1^	-	1.01 (0.67, 1.51)	0.98	-	1.09 (0.69, 1.72)	0.73	-
Excessive drinking ^1^	-	1.06 (0.72, 1.56)	0.77	-	0.85 (0.55, 1.31)	0.46	-
Occupation ^1^	-	0.24 (0.06, 1.00)	0.05	-	0.28 (0.07, 1.21)	0.09	-
Newly diagnosed ^1^	-	2.56 (1.55, 4.23)	<0.001	-	2.18 (1.27, 3.74)	0.005	-
Exposure to sunshine ^1^	-	1.73 (1.11, 2.69)	0.02	-	1.88 (1.17, 3.02)	0.009	-
Vitamin A (ug RAE/d) ^3^	<147.5	0.77 (0.50,1.18)	0.23	0.14	0.71 (0.44, 1.14)	0.15	0.12
	147.5–346.0	0.68 (0.44, 1.07)	0.09		0.72 (0.44, 1.16)	0.17	
	≥346.0	-	-		-		
Thiamine (mg/d)	<0.8	1.15 (0.73, 1.81)	0.56	0.9	1.22 (0.60, 2.47)	0.59	0.58
	0.8–1.3	1.10 (0.71, 1.71)	0.66		1.03 (0.57, 1.86)	0.92	
	≥1.3	-	-		-		
Nicotinic acid (mg NE/d) ^3^	<9.5	1.19 (0.76, 1.85)	0.45	0.37	0.72 (0.36, 1.43)	0.35	0.32
	9.5–13.6	0.98 (0.62, 1.56)	0.94		0.82 (0.48, 1.41)	0.47	
	≥13.6	-	-		-		
Riboflavin (mg/d)	<0.5	0.68 (0.34, 1.36)	0.27	0.14	0.69 (0.34, 1.39)	0.30	0.28
	0.5–0.7	0.85 (0.49, 1.48)	0.56		0.85 (0.49, 1.49)	0.57	
	≥0.7	-	-		-		
Vitamin C (mg/d)	<32.0	0.87 (0.54, 1.39)	0.56	0.19	1.80 (1.07, 3.04)	0.03	0.02
	32.0–70.0	1.18 (0.76, 1.82)	0.46		1.39 (0.85, 2.29)	0.19	
	≥70.0	-	-		-		
Vitamin E (mg α-TE/d) ^3^	<8.6	1.55 (0.99, 2.42)	0.05	0.05	1.23 (0.59, 2.54)	0.58	0.69
	8.6–15.2	1.06 (0.66, 1.71)	0.80		0.99 (0.58, 1.70)	0.98	
	≥15.2	-	-		-		
K (mg/d)	<1229.4	1.20 (0.77, 1.86)	0.43	0.41	1.29 (0.55, 3.02)	0.56	0.55
	1229.4–1731.4	1.00 (0.63, 1.59)	0.99		1.20 (0.67, 2.17)	0.54	
	≥1731.4	-	-		-		
Na (mg/d) ^4^	<249.9	1.09 (0.68, 1.74)	0.72	0.45	1.48 (0.90, 2.43)	0.12	0.19
	249.9–716.0	1.39 (0.89, 2.18)	0.15		1.05 (0.64, 1.71)	0.86	
	≥716.0	-	-		-		
Ca (mg/d)	<211.0	1.29 (0.83, 2.00)	0.26	0.23	1.02 (0.54, 1.91)	0.96	0.95
	211.0–340.5	1.00 (0.63, 1.59)	0.99		0.91 (0.54, 1.53)	0.72	
	≥340.5	-	-		-		
Mg (mg/d)	<240.0	1.54 (1.00, 2.39)	0.05	0.02	0.33 (0.14, 0.79)	0.01	0.01
	240.0–354.0	0.93 (0.58, 1.51)	0.78		0.50 (0.28, 0.90)	0.02	
	≥354.0	-	-		-		
P (mg/d)	<801.0	1.58 (1.01, 2.47)	0.05	0.1	0.75 (0.28, 2.02)	0.56	0.43
	801.0–1116.0	1.13 (0.70, 1.82)	0.62		0.59 (0.30, 1.15)	0.12	
	≥1116.0	-	-		-		
Fe (mg/d)	<14.6	1.10 (0.72, 1.69)	0.66	0.13	0.90 (0.33, 2.44)	0.84	0.84
	14.6–20.4	0.77 (0.48, 1.22)	0.26		1.35 (0.70, 2.60)	0.37	
	≥20.4	-	-		-		
Zn (mg/d)	<7.2	1.17 (0.74, 1.85)	0.49	0.9	2.52 (1.25, 5.08)	0.01	0.02
	7.2–10.2	1.15 (0.73, 1.80)	0.55		1.29 (0.76, 2.19)	0.34	
	≥10.2	-	-		-		
Cu (mg/d)	<1.4	1.41 (0.91, 2.19)	0.12	0.01	0.48 (0.22, 1.02)	0.06	0.10
	1.4–2.0	0.81 (0.49, 1.34)	0.41		0.77 (0.45, 1.32)	0.34	
	≥2.0	-	-		-		
Se (μg/d)	<30.6	1.46 (0.92, 2.31)	0.11	0.67	1.05 (0.61, 1.83)	0.86	0.88
	30.6–44.5	1.36 (0.85, 2.17)	0.20		0.77 (0.46, 1.29)	0.32	
	≥44.5	-	-		-		
Mn (mg/d)	<4.6	1.19 (0.77, 1.82)	0.43	0.05	0.71 (0.33, 1.51)	0.37	0.44
	4.6–6.8	0.75 (0.46, 1.21)	0.23		0.86 (0.50, 1.50)	0.61	
	≥6.8	-	-		-		

^1^ The female, non-smoking, non-excessive drinking, farmers, newly diagnosed and exposure to sunshine for >40 min group were used as the reference group. ^2^ The multivariate model was adjusted for confounding factors including the age, gender, BMI, smoking, excessive drinking, occupation, newly or relapsed tuberculosis, presence of lung cavity, diabetes and exposure to sunshine, as well as the intakes of vitamin A, vitamin C, vitamin E, Zn, Mg and Cu. ^3^ 1 ug RAE = 1 ug all-trans-retinol + (1/12) ug all-trans-β-carotene + (1/24) ug provitamin A carotenoid; 1 mg NE = 1 mg niacin + (1/60) mg tryptophan; 1 mg α-TE = 1 mg α-tocopherol + 0.5 mg β-tocopherol + 0.1 mg γ-tocopherol + 0.02 mg δ-tocopherol + 0.3 mg α-tocotrienol. ^4^ The intake of Na as salt was not counted.

**Table 3:** Association of the dietary micronutrient intake with the TB score^1^ at the end of Week 2 antituberculosis treatment.

	Group	Univariate			Multivariate ^2^		
		OR (95% CI)	P	P_trend_	OR (95% CI)	P	P_trend_
Age	-	1.02 (1.01, 1.02)	<0.001	-	1.01 (1.00, 1.02)	0.01	-
Gender ^3^	-	0.76 (0.61, 0.95)	0.02	-	0.80 (0.62, 1.04)	0.09	-
BMI	-	0.90 (0.87, 0.93)	<0.001	-	0.91 (0.87, 0.94)	<0.001	-
Smoking ^3^	-	1.11 (0.91, 1.37)	0.31	-	0.90 (0.70, 1.14)	0.38	-
Excessive drinking ^3^	-	3.78 (3.07, 4.65)	<0.001	-	3.10 (2.46, 3.89)	<0.001	-
Occupation ^3^		0.54 (0.36, 0.82)	0.003	-	0.44 (0.28, 0.70)	0.001	-
Newly diagnosed ^3^		1.79 (1.27, 2.52)	0.001	-	1.60 (1.10, 2.33)	0.02	-
Exposure to sunshine ^3^		2.90 (2.20, 3.81)	<0.001	-	2.40 (1.78, 3.24)	<0.001	-
Vitamin A (ug RAE/d) ^4^	<147.5	0.82 (0.65, 1.02)	0.08	0.11	0.64 (0.49, 0.83)	0.001	0.002
	147.5–346.0	0.96 (0.77,1.20)	0.71		0.90 (0.70, 1.16)	0.42	
	≥346.0	-	-		-		
Thiamine (mg/d)	<0.8	0.78 (0.62, 0.99)	0.04	0.08	0.84 (0.57, 1.24)	0.38	0.48
	0.8–1.3	0.55 (0.44, 0.69)	<0.001		0.72 (0.53, 0.98)	0.04	
	≥1.3	-	-		-		
Nicotinic acid (mg NE/d) ^4^	<9.5	0.88 (0.70, 1.10)	0.27	0.17	0.59 (0.41, 0.86)	0.01	0.01
	9.5–13.6	0.75 (0.60, 0.94)	0.01		0.87 (0.65, 1.16)	0.34	
	≥13.6	-	-		-		
Riboflavin (mg/d)	<0.5	0.67 (0.54, 0.85)	0.001	0.001	0.61 (0.42, 0.88)	0.01	0.002
	0.5–0.7	0.57 (0.45, 0.74)	<0.001		0.67 (0.50, 0.91)	0.01	
	≥0.7	-	-		-		
Vitamin C (mg/d)	<32.0	1.90 (1.52, 2.39)	<0.001	<0.001	1.49 (1.14, 1.96)	0.004	0.002
	32.0–70.0	1.56 (1.24, 1.96)	<0.001		1.32 (1.02, 1.71)	0.04	
	≥70.0	-	-		-		
Vitamin E (mg α-TE/d) ^4^	<8.6	1.12 (0.89, 1.40)	0.33	0.59	0.86 (0.59, 1.26)	0.45	0.27
	8.6–15.2	0.85 (0.68, 1.07)	0.17		0.83 (0.62, 1.11)	0.21	
	≥15.2	-	-		-		
K (mg/d)	<1229.4	1.10 (0.88, 1.37)	0.42	0.48	0.61 (0.39, 0.95)	0.03	0.03
	1229.4–1731.4	0.89 (0.71, 1.11)	0.29		0.85 (0.61, 1.18)	0.32	
	≥1731.4	-	-		-		
Na (mg/d) ^5^	<249.9	1.19 (0.95, 1.50)	0.12	0.09	0.91 (0.70, 1.19)	0.50	0.56
	249.9–716.0	1.18 (0.94, 1.48)	0.15		0.92 (0.72, 1.19)	0.54	
	≥716.0	-	-		-		
Ca (mg/d)	<211.0	1.48 (1.18, 1.86)	0.001	0.001	1.34 (0.96, 1.88)	0.08	0.10
	211.0–340.5	1.14 (0.91, 1.43)	0.26		1.17 (0.88, 1.55)	0.28	
	≥340.5	-	-		-		
Mg (mg/d)	<240.0	1.41 (1.13, 1.77)	0.003	0.004	2.07 (1.32, 3.23)	0.001	0.001
	240.0–354.0	1.04 (0.83, 1.30)	0.73		1.50 (1.09, 2.07)	0.01	
	≥354.0	-	-		-		
P (mg/d)	<801.0	1.10 (0.88, 1.37)	0.42	0.47	1.37 (0.81, 2.30)	0.24	0.17
	801.0–1116.0	0.90 (0.72, 1.13)	0.38		1.26 (0.87, 1.83)	0.22	
	≥1116.0	-	-		-		
Fe (mg/d)	<14.6	1.16 (0.92, 1.45)	0.21	0.26	1.48 (0.88, 2.49)	0.14	0.13
	14.6–20.4	0.83 (0.66, 1.04)	0.11		1.12 (0.77, 1.62)	0.57	
	≥20.4	-	-		-		
Zn (mg/d)	<7.2	0.85 (0.68, 1.06)	0.16	0.12	0.50 (0.35, 0.73)	<0.001	<0.001
	7.2–10.2	0.62 (0.50, 0.78)	<0.001		0.57 (0.43, 0.76)	<0.001	
	≥10.2	-	-		-		
Cu (mg/d)	<1.4	0.95 (0.77, 1.19)	0.67	0.51	0.80 (0.54, 1.18)	0.26	0.39
	1.4–2.0	0.73 (0.58, 0.92)	0.006		0.82 (0.60, 1.11)	0.19	
	≥2.0	-	-		-		
Se (μg/d)	<30.6	1.00 (0.80, 1.25)	0.98	0.95	1.02 (0.75, 1.39)	0.90	0.98
	30.6–44.5	0.95 (0.76, 1.18)	0.63		1.04 (0.80, 1.36)	0.77	
	≥44.5	-	-		-		
Mn (mg/d)	<4.6	1.50 (1.20, 1.88)	<0.001	<0.001	3.03 (2.01, 4.55)	<0.001	<0.001
	4.6–6.8	1.29 (1.03, 1.62)	0.03		2.17 (1.57, 2.99)	<0.001	
	≥6.8	-	-		-		

^1^ TB score was used as a comprehensive index to assess the clinical symptoms of the participants, as reported previously (reference 12). Having any of the eight mentioned symptoms above would give one point; having a BMI equal or less than 16 kg/m^2^ would give two points, BMI between 16 kg/m^2^ and 18 kg/m^2^ would give one point, and BMI more than 18 kg/m^2^ would give zero point. Therefore, TB score would range from 0 to 10. A higher TB score indicates a more severe clinical symptom. The TB score results were divided into two groups with a close number of participants (TB score ≤3 or TB score >3 group). ^2^ The multivariate model was adjusted for confounding factors, including age, gender, BMI, smoking, excessive drinking, occupation, newly or relapsed tuberculosis, presence of lung cavity, diabetes and exposure to sunshine, as well as the intakes of vitamin A, vitamin C, vitamin E, Zn, Mg and Cu. ^3^ The female, non-smoking, non-excessive drinking, farmers, newly diagnosed and exposure to sunshine for >40 min group were used as the reference group. ^4^ 1 ug RAE = 1 ug all-trans-retinol + (1/12) ug all-trans-β-carotene + (1/24) ug provitamin A carotenoid; 1 mg NE = 1 mg niacin + (1/60) mg tryptophan; 1 mg α-TE = 1 mg α-tocopherol + 0.5 mg β-tocopherol + 0.1 mg γ-tocopherol + 0.02 mg δ-tocopherol + 0.3 mg α-tocotrienol. ^5^ The intake of Na as salt was not counted.

## References

[1] World Health Organization (2019). Global Tuberculosis Report 2019.

[2] Dheda K., Barry C.E., Maartens G. (2016). Tuberculosis. Lancet.

[3] Hoyt K.J., Sarkar S., White L., Joseph N.M., Salgame P., Lakshminarayanan S., Muthaiah M., Vinod Kumar S., Ellner J.J., Roy G. (2019). Effect of malnutrition on radiographic findings and mycobacterial burden in pulmonary tuberculosis. PLoS ONE.

[4] Chandrasekaran P., Saravanan N., Bethunaickan R., Tripathy S. (2017). Malnutrition: Modulator of Immune Responses in Tuberculosis. Front. Immunol..

[5] Estrella J.L., Kan-Sutton C., Gong X., Rajagopalan M., Lewis D.E., Hunter R.L., Eissa N.T., Jagannath C. (2011). A Novel In Vitro Human Macrophage Model to Study the Persistence of Mycobacterium tuberculosis Using Vitamin D(3) and Retinoic Acid Activated THP-1 Macrophages. Front. Microbiol..

[6] Yuk J.-M., Shin D.-M., Lee H.-M., Yang C.-S., Jin H.S., Kim K.-K., Lee Z.-W., Lee S.-H., Kim J.-M., Jo E.-K. (2009). Vitamin D3 Induces Autophagy in Human Monocytes/Macrophages via Cathelicidin. Cell Host Microbe.

[7] Soh A.Z., Chee C.B.E., Wang Y.T., Yuan J.M., Koh W.P. (2017). Dietary Intake of Antioxidant Vitamins and Carotenoids and Risk of Developing Active Tuberculosis in a Prospective Population-Based Cohort Study. Am. J. Epidemiol..

[8] Fox G.J., Lee R.S., Lucas M., Khan F.A., Proulx J.-F., Hornby K., Jung S., Benedetti A., Behr M.A., Menzies D. (2015). Inadequate Diet Is Associated with Acquiring Mycobacterium tuberculosis Infection in an Inuit Community. A Case–Control Study. Ann. Am. Thorac. Soc..

[9] Hemilä H., Kaprio J. (2008). Vitamin E supplementation may transiently increase tuberculosis risk in males who smoke heavily and have high dietary vitamin C intake. Br. J. Nutr..

[10] Lewandowska M., Więckowska B., Sajdak S., Lubiński J. (2020). First Trimester Microelements and their Relationships with Pregnancy Outcomes and Complications. Nutrients.

[11] Tam E., Keats E.C., Rind F., Das J.K., Bhutta A.Z.A. (2020). Micronutrient Supplementation and Fortification Interventions on Health and Development Outcomes among Children Under-Five in Low- and Middle-Income Countries: A Systematic Review and Meta-Analysis. Nutrients.

[12] Rowe S., Carr A.C. (2020). Global Vitamin C Status and Prevalence of Deficiency: A Cause for Concern?. Nutrients.

[13] Carr A.C., Maggini S. (2017). Vitamin C and Immune Function. Nutrients.

[14] Chasapis C.T., Ntoupa P.A., Spiliopoulou C.A., Stefanidou M.E. (2020). Recent aspects of the effects of zinc on human health. Arch. Toxicol..

[15] Xiao D., Bureau of Disease Control and Prevention, Ministry of Health of China (2008). The Chinese National Tuberculosis Prevention and Control Guideline.

[16] Lin S., Gao T., Sun C., Jia M., Liu C., Ma X., Ma A. (2019). Association of dietary patterns and endoscopic gastric mucosal atrophy in an adult Chinese population. Sci. Rep..

[17] Yang Y., Institute of Nutrition and Health, Chinese Center for Disease Control and Prevention (2018). China Food Composition Tables.

[18] Wejse C., Gustafson P., Nielsen J., Gomes V.F., Aaby P., Andersen P.L., Sodemann M. (2008). TBscore: Signs and symptoms from tuberculosis patients in a low-resource setting have predictive value and may be used to assess clinical course. Scand. J. Infect. Dis..

[19] Karyadi E., West C.E., Schultink W., Nelwan R.H., Gross R., Amin Z., Dolmans W.M., Schlebusch H., van der Meer J.W. (2002). A double-blind, placebo-controlled study of vitamin A and zinc supplementation in persons with tuberculosis in Indonesia: Effects on clinical response and nutritional status. Am. J. Clin. Nutr..

[20] Jaganath D., Mupere E. (2012). Childhood tuberculosis and malnutrition. J. Infect. Dis..

[21] Box G.E.P., Tidwell P.W. (1962). Transformation of the Independent Variables. Technometrics.

[22] Chinese Nutrition Society (2013). Chinese Dietary Reference Intakes.

[23] Vilcheze C., Hartman T., Weinrick B., Jacobs W.R. (2013). Mycobacterium tuberculosis is extraordinarily sensitive to killing by a vitamin C-induced Fenton reaction. Nat. Commun..

[24] Vilchèze C., Kim J., Jacobs W.R. (2018). Vitamin C Potentiates the Killing of Mycobacterium tuberculosis by the First-Line Tuberculosis Drugs Isoniazid and Rifampin in Mice. Antimicrob. Agents Chemother..

[25] Pakasi T.A., Karyadi E., Suratih N.M.D., Salean M., Darmawidjaja N., Bor H., van der Velden K., Dolmans W.M.V., van der Meer J.W.M. (2010). Zinc and vitamin A supplementation fails to reduce sputum conversion time in severely malnourished pulmonary tuberculosis patients in Indonesia. Nutr. J..

[26] Montgomery A.A., Peters T.J., Little P. (2003). Design, analysis and presentation of factorial randomised controlled trials. BMC Med. Res. Methodol..

[27] Seyedrezazadeh E., Ostadrahimi A., Mahboob S., Assadi Y., Ansarin K., Shakoori P., Pourmoghaddam M. (2006). Vitamin E-Selenium Supplement and Clinical Responses of Active Pulmonary Tuberculosis. Tanaffos.

[28] Neyrolles O., Wolschendorf F., Mitra A., Niederweis M. (2015). Mycobacteria, metals, and the macrophage. Immunol. Rev..

[29] Grobler L., Nagpal S., Sudarsanam T.D., Sinclair D. (2016). Nutritional supplements for people being treated for active tuberculosis. Cochrane Database Syst. Rev..

[30] Wang J., Xiong K., Wang Q., Zhao S., Liu Y., Ma A. (2020). Adjunctive vitamin A and D during pulmonary tuberculosis treatment: A randomized controlled trial with a 2 × 2 factorial design. Food Funct..

[31] Saraff V., Shaw N. (2016). Sunshine and vitamin D. Arch. Dis. Child.

[32] Carr A.C., Rowe S. (2020). Factors Affecting Vitamin C Status and Prevalence of Deficiency: A Global Health Perspective. Nutrients.

